# Flaws in Estrus Synchronization Protocols Increase Vaginal Prolapse and Hydrometra Risk in Sheep

**DOI:** 10.3390/life15050795

**Published:** 2025-05-17

**Authors:** Nikolaos Tsekouras, Ioannis Tsakmakidis, Dimitrios Gougoulis, Mathis A. B. Christodoulopoulos, Christos Kousoulis, Georgios I. Papakonstantinou, Vasileios G. Papatsiros, Georgios Christodoulopoulos

**Affiliations:** 1 Clinic of Medicine, Faculty of Veterinary Medicine, University of Thessaly, 43100 Karditsa, Greece; nitsekou@vet.uth.gr (N.T.); dgoug@uth.gr (D.G.); geopapak@vet.uth.gr (G.I.P.); vpapatsiros@vet.uth.gr (V.G.P.); 2 Department of Agriculture, School of Agricultural Sciences, University of Western Macedonia, Kontopoulou str, 53100 Florina, Greece; itsak@m-t.gov.gr; 3 Faculty of Sciences, Aix-Marseille University, 13013 Marseille, France; mathis.christodoulopoulos@etu.univ-amu.fr; 4 Agricultural Cooperative of Cow and Sheep Farmers of Western Thessaly, 43060 Karditsa, Greece; kouschristos@uth.gr; 5 Department of Animal Science, Agricultural University of Athens, 11855 Athens, Greece

**Keywords:** vaginal prolapse, hydrometra, reproductive management, intensive sheep farming

## Abstract

This study examines the reproductive outcomes of Lacaune-crossbred ewes and hoggets in intensive production systems, focusing on vaginal prolapse and hydrometra associated with flaws in estrus synchronization (E.S.) protocols. Data from multiple farms were combined for analysis due to the absence of significant variation at the farm level. The findings revealed a strong association between vaginal prolapse, parity, and litter size, with hoggets carrying multiple fetuses facing the highest risk (*p* < 0.0001). This highlights the need to reconsider equine chorionic gonadotropin (eCG) administration in hoggets, as it increases the likelihood of multiple pregnancies and, consequently, prolapse. Additionally, a progressive rise in hydrometra prevalence was observed with repeated synchronization cycles in ewes, increasing from 0.51% after the third treatment to 12.33% after the fourth (*p* < 0.0001). Notably, in this study, the four synchronization cycles were applied over a relatively short period (7.22 ± 1.64 months), further supporting concerns that excessive hormonal treatments within a compressed timeframe exacerbate reproductive dysfunction. The results corroborate previous reports that prolonged progesterone exposure can impair uterine function, leading to fluid retention and hydrometra. To mitigate these risks, estrus synchronization protocols should be critically reassessed—especially by extending the interval between successive treatments—to protect reproductive health and animal welfare. These findings not only underscore the need for more welfare-conscious practices in intensive sheep farming but also encourage further research aimed at refining hormonal management strategies in dairy ewe reproduction.

## 1. Introduction

Sheep play a vital role in global agricultural systems by supporting food security, rural livelihoods, and sustainable production. Compared to other livestock species such as cattle and pigs, sheep are recognized for their relatively low ecological footprint, making them a more environmentally sustainable choice [1,2,3]. According to the Food and Agriculture Organization (FAO), the global sheep population exceeds 1 billion animals [4]. In Greece, where this study was conducted, sheep farming is a major component of the agricultural economy, with approximately 7.3 million sheep distributed across around 83,000 holdings [5].

Sheep production in Greece is primarily focused on dairy, driven by the demand for traditional products such as feta cheese and sheep-milk yogurt [6,7]. In recent years, the sector has experienced considerable restructuring. Although the overall number of animals has declined, there has been a notable increase in the number of large-scale, intensively managed farms, which typically maintain over 500 ewes [8,9].

Within these intensive production systems, estrus synchronization has become a widely adopted reproductive management practice. The most commonly used protocol involves intravaginal progestagen sponges in combination with the intramuscular administration of equine chorionic gonadotropin (eCG), inducing estrus during the seasonal anestrus period (January to March) [10]. In Greece, natural reproductive activity typically resumes in April and peaks in May. Estrus synchronization, besides ensuring a steady milk supply, also facilitates synchronized lambings and enables more efficient labor organization [11,12]. Although hormonal protocols offer substantial production benefits, they may also introduce health and welfare challenges—particularly in high-input, high-output systems [13]. Among the reproductive disorders observed in intensive sheep farming are vaginal prolapse and hydrometra.

Vaginal prolapse involves the eversion and protrusion of the vaginal wall through the vulva, most commonly occurring during late gestation or postpartum, but can affect non-pregnant animals as well [14,15,16,17,18]. It is particularly prevalent in sheep, with risk factors including multiple fetuses, high parity, and genetic predisposition [19,20]. Prolapse recurrence is common, and culling is often recommended [21,22,23]. Left untreated, it can lead to severe complications such as tissue necrosis, uterine rupture, and abdominal organ herniation, resulting in animal suffering and economic loss [21,24,25,26,27]. Hydrometra (pseudopregnancy) is another concern, characterized by persistent anestrus and abdominal enlargement without fetal development, and is often linked to hormonal treatments [28,29,30,31,32,33,34]. It affects reproductive efficiency and milk production [35,36].

In Greece, field veterinarians and producers have recently noted an apparent increase in cases of vaginal prolapse among young animals and hydrometra in adult ewes following synchronization protocols. However, these observations are anecdotal, and no comprehensive epidemiological studies have been conducted to date.

This study aims to assess the prevalence and risk factors of vaginal prolapse and hydrometra in intensively managed dairy sheep flocks in Greece, with a particular focus on the impact of estrus synchronization. The findings highlight the need for a critical reassessment of current reproductive practices and their frequency in intensive production systems. Additionally, they stress the importance of further research to refine synchronization protocols and hormone dosages, with the aim of fostering more sustainable and ethically sound management. The results offer practical value for flock-level decision-making, veterinary care, and the reduction in economic losses and animal welfare concerns linked to these disorders.

## 2. Materials and Methods

### 2.1. Study Design

A cross-sectional observational study was conducted between 1 September 2022 and 29 February 2024 in the counties of Karditsa, Trikala, and Larissa, located in the Thessaly region of Central Greece. This area is a major hub for intensive dairy sheep farming, representing typical conditions of large-scale, specialized milk production systems in the country [5].

A total of 140 commercial dairy sheep farms participated in the study. The combined number of reproductive females (ewes and hoggets) across all farms was 97,215, with individual flock sizes ranging from 500 to 1400 animals. These figures align with the structural profile of modern Greek dairy sheep enterprises [7].

### 2.2. Farm Selection Criteria

Farms were enrolled in the study based on the following inclusion criteria:

(i) Housing system: Exclusive use of fully indoor intensive housing, characterized by permanent confinement and feeding with total mixed rations composed of commercial concentrates and locally produced forages (hay and/or ensilage).
(ii) Flock size: A minimum of 500 reproductive females, corresponding to the economic threshold for viability in intensive Greek dairy sheep operations [5].
(iii) Estrus synchronization protocols: Routine application of hormonal estrus synchronization using progestagen-impregnated intravaginal sponges (inserted for 12–14 days), followed by an intramuscular injection of 500 IU of eCG at sponge removal [7].
(iv) Ultrasonographic pregnancy diagnosis: Regular use of transabdominal ultrasonography between 45 and 60 days post-mating, as part of standard reproductive monitoring procedures.

All participating farmers provided written informed consent prior to inclusion in the study.

### 2.3. Animals

The sheep population involved in the study consisted predominantly of Lacaune-crossbred animals, which constitute the dominant genotype in intensively managed dairy sheep farms across Central Greece.

In the modern Greek sheep industry, the Lacaune-crossbred genotype has developed through the systematic crossing of French Lacaune rams with indigenous Greek ewes, primarily of the Karagouniko and Chios breeds, and to a lesser extent with other local sheep populations. The crossing programs were initiated to improve milk production while retaining the adaptive traits of the local breeds. The resulting Lacaune-crossbred animals have demonstrated good adaptation to intensive farming conditions and are now widely established in large-scale dairy operations throughout the country [8,9]. Morphologically, they closely resemble the Lacaune breed in terms of body size and udder development, although some phenotypic variation persists—such as slightly more extensive wool coverage and occasional black facial spots—reflecting their local genetic background. It should be noted that there is currently no systematic zootechnical description of the Greek Lacaune crossbreed in the literature.

### 2.4. Data Collection

#### 2.4.1. Reproductive History

Reproductive management records were systematically compiled for each farm throughout the study period. Collected data included:

- Annual milk production per flock;
- Age group classification of reproductive females (hoggets or ewes);
- Dates of estrus synchronization procedures and ram introduction for natural mating.

#### 2.4.2. Clinical Examination

Biweekly farm visits were conducted over the entire study duration. During each visit, all pregnant animals were examined for clinical signs of vaginal prolapse. Visual inspection was performed first, followed by manual palpation if needed to confirm diagnosis. Vaginal prolapse was defined as partial or complete eversion of the vaginal wall through the vulva, with or without cervical involvement, in accordance with standard diagnostic criteria [23].

#### 2.4.3. Ultrasonographic Examination

Ultrasound scans were performed using a portable ultrasound unit equipped with a 3.5–5.0 MHz convex transducer, following established protocols for pregnancy diagnosis in sheep [13]. Scanning was conducted between 45 and 70 days post-mating.

- Fetal numbers were determined via ultrasound and later confirmed or corrected based on parturition outcomes or post-mortem findings in cases of pregnancy loss.
- Hydrometra diagnosis was based on specific ultrasonographic criteria [37,38]:
  (i) Presence of anechoic or hypoechoic intrauterine fluid;
  (ii) Absence of fetal or embryonic structures;
  (iii) Visualization of fine echogenic septations within the fluid, when present.

### 2.5. Data Management and Statistical Analysis

All data were collected through structured on-site interviews with farmers, combined with a review of farm records and direct observation during farm visits. Standardized data collection sheets were used to ensure consistency across all 140 participating farms. Information was recorded on flock demographics, reproductive protocols, clinical cases of vaginal prolapse and hydrometra, and management practices.

Collected data were then entered into a custom digital database using Microsoft Excel (Microsoft Corp., Redmond, WA, USA) and cross-checked for accuracy and completeness by a second researcher.

- The prevalence of vaginal prolapse and hydrometra was calculated for the entire study population.
- Associations between categorical variables (e.g., hoggets vs. ewes) were analyzed using the Chi-square (χ^2^) test.
- Differences among multiple groups (e.g., based on the number of estrus synchronization applications or parity) were assessed using one-way analysis of variance (ANOVA).
- A *p*-value < 0.05 was considered statistically significant.

All statistical analyses were conducted using SPSS software, version 28.0 (IBM Corp., Armonk, NY, USA).

## 3. Results

### 3.1. Study Population Overview

The study population consisted of Lacaune-crossbred ewes and hoggets, with farm breeding records indicating an estimated Karagouniko breed ancestry of less than 20%. These animals exhibited the morphological traits of the Lacaune breed. Adult females had an average body weight of approximately 70.0 ± 4.1 kg and a mean annual milk production of 420.0 ± 42.0 kg during an 8-month lactation period (n = 85,403).

Hoggets were first introduced to intravaginal sponges at an average age of 8.5 ± 1.05 months (n = 14,494). All animals followed the same estrus synchronization protocol, which involved the insertion of intravaginal sponges impregnated with 60 mg of Medroxyprogesterone Acetate (OVIGEST^®^; LABORATORIOS HIPRA, S.A. Avda. la Selva, 135. 17170-Amer (Girona) Spain) for 14 days. Following sponge removal, an intramuscular injection of 500 IU of eCG (GONASER^®^ 5000 IU; LABORATORIOS HIPRA, S.A. Avda. la Selva, 135. 17170-Amer (Girona) Spain) was administered.

The population included hoggets as well as ewes in their second, third, and fourth parities, with one lambing per year. In the intensive production system applied, ewes were typically retained until their fourth lambing and were culled at the end of the following lactation period (Table 1).

### 3.2. Vaginal Prolapse

**Figure 1:**
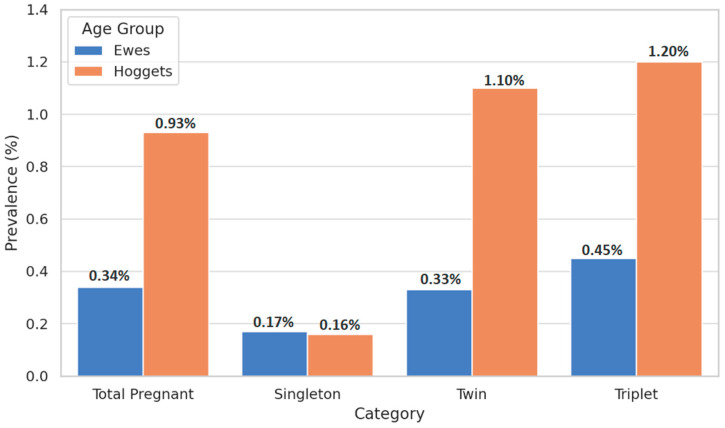
Prevalence of vaginal prolapse in ewes and hoggets across pregnancy categories.

A higher risk of vaginal prolapse was confirmed in cases of multiple pregnancies. The incidence of vaginal prolapse was evaluated in hoggets during their first pregnancy and compared with ewes at their second, third, or fourth parity. As shown in Table 2 and Figure 1, vaginal prolapse occurred significantly more frequently in hoggets than in ewes across all litter sizes (*p* < 0.0001). However, in single pregnancies, the percentage of vaginal prolapse in hoggets was arithmetically lower than in ewes. Additionally, among both hoggets and ewes, a progressive increase in prolapse prevalence was observed as litter size increased. The highest incidence of vaginal prolapse was recorded in triplet-bearing females (*p* < 0.0001).

### 3.3. Hydrometra

Hydrometra was detected only after repeated hormonal treatments. The analysis of hydrometra was restricted to ewes in their first or second parity. In accordance with standard farm policy, ewes with three lambings that failed to conceive after a single synchronization attempt were culled, while hoggets were removed from the herd after two unsuccessful attempts.

**Figure 2:**
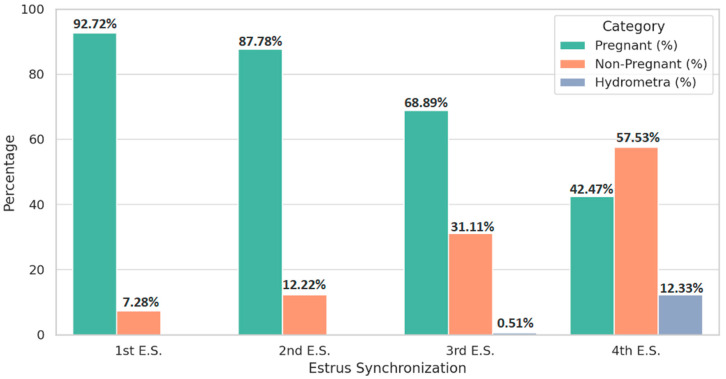
Pregnancy outcomes and hydrometra prevalence across the four estrus synchronization protocols.

Table 3 and Figure 2 present the prevalence of hydrometra following successive estrus synchronizations. A total of 146 ewes received four synchronization treatments over an average period of 7.22 ± 1.64 months. The prevalence of hydrometra rose significantly with the number of synchronization attempts (*p* < 0.0001). No cases were detected after the first or second treatment; however, 0.51% of animals developed hydrometra after the third synchronization, increasing markedly to 12.33% after the fourth.

## 4. Discussion

Reproductive disorders in sheep may result in considerable economic losses for farmers, largely due to compromised fertility, reduced productive lifespan, prolonged lambing intervals, diminished conception rates, and increased veterinary interventions [39,40]. Among the most commonly encountered reproductive problems in sheep are vaginal prolapse and hydrometra [41], both of which may be exacerbated in intensive farming systems employing estrus synchronization protocols.

The global incidence of vaginal prolapse in ruminants varies widely. Reported rates range from 0.21% to 22.5% in buffaloes [42,43,44], 0.51% to 11.3% in cattle [45,46,47,48,49], and 3.6% to 31.25% in goats [39,50,51]. In sheep, prevalence is typically between 0.1% and 2.2%, although isolated flock-level outbreaks can reach rates exceeding 15% and in rare instances surpass 40% [24,27,52,53,54,55,56,57,58].

Multiple predisposing factors have been implicated in the etiology of vaginal prolapse. Endocrine alterations during the final trimester of pregnancy—specifically, elevated estrogen concentrations and relaxin production—can induce marked relaxation and edema of pelvic ligaments and perivaginal soft tissues. This physiological relaxation, when combined with increased intra-abdominal pressure due to the gravid uterus, is considered the primary mechanistic basis for prolapse [22,59,60,61,62]. Additional contributors to intra-abdominal pressure include excessive visceral fat, ruminal distension, and multiparity [63,64]. The impact of fetal number is particularly important: a New Zealand study observed that the risk of vaginal prolapse was 5.3 times higher in twin pregnancies and 11.3 times higher in triplet pregnancies compared to singletons [27].

Environmental and nutritional factors also contribute to the problem. Exposure to extreme cold, lack of exercise, previous vaginal injuries, poor anatomical structure, and diets rich in phytoestrogens—such as those based on clover, soybean meal, or moldy grains—have all been linked to a higher risk of prolapse. Deficiencies in essential macro- and micronutrients may also increase the risk [17,65,66,67,68,69,70,71]. Furthermore, specific structural aspects of animal housing, including sloped lambing paddocks and elevated feed bunks, have been suggested as mechanical contributors to prolapse [27,72,73].

Vaginal prolapse is clinically classified into four grades, with Grade I being intermittent and often only visible when the ewe is recumbent, and Grade IV involving chronic prolapse with secondary trauma, infection, or necrosis [74]. In our clinical approach, due to the large number of animals being examined, initial inspection was conducted visually, and only animals showing visible abnormalities were subjected to further manual examination. As a result, it is possible that some early-stage cases, particularly those corresponding to Grade I, may have been missed during initial visits. However, because farm visits were conducted on a biweekly basis, any previously unnoticed cases were likely to be identified during subsequent inspections. This repeated monitoring increases the reliability of the total number of cases recorded.

In the present study, a statistically significant association was observed between vaginal prolapse and both parity and litter size, with the highest risk recorded in hoggets carrying multiple fetuses (*p* < 0.0001). These findings are in line with previous reports linking increased fetal load in younger animals with elevated intra-abdominal strain and consequent prolapse. The heightened susceptibility in hoggets may stem from their underdeveloped musculoskeletal systems, which are potentially inadequate for the physical demands of twin or triplet gestation [27,73].

In Greece, it is generally recommended that hoggets be bred only when they have reached at least two-thirds of their mature body weight (approximately 70 kg) or are at least nine months of age. Nonetheless, these guidelines are often not adhered to in practice. Breeding underweight or physiologically immature animals increases the likelihood of obstetric complications, including vaginal prolapse, due to inadequate pelvic development [23,27]. Moreover, the administration of standard adult doses of eCG to underweight hoggets may result in supraphysiological gonadotropic stimulation per kilogram of body mass, thereby increasing the probability of multiple ovulations and conceptions—and, consequently, the risk of prolapse. A revision of current synchronization protocols, particularly with respect to eCG dosing in young or light-weight ewes, is warranted.

Regarding hydrometra, incidence rates in small ruminants are similarly variable. In goats, reported prevalence ranges from 0.4% to 16% and in certain cases nearly 51% [36,75,76,77,78]. In sheep, rates typically range between 0.15% and 4.70% [18,79,80,81,82,83], although levels up to 10% have also been reported [36].

The pathophysiology of hydrometra is closely associated with altered luteal function and disrupted luteolysis, often linked to excessive or repeated exposure to exogenous hormones. Prolactin, a hormone with luteotropic action in pseudopregnant animals, has been implicated in hydrometra development, particularly in cases of sustained corpus luteum activity [84]. Several risk factors have been described, including increasing age, poor body condition, prior reproductive failure, rising parity, hormonal estrus induction, and even the presence of domestic carnivores in the farm environment, possibly serving as vectors of embryotoxic pathogens [36,38,85,86]. In sheep, estrus synchronization has been identified as a consistent risk factor [33,34].

In our study, no hydrometra cases were observed after the first or second synchronization cycle. However, prevalence rose to 0.51% following the third treatment and escalated dramatically to 12.33% after the fourth cycle (*p* < 0.0001). Notably, these synchronization rounds occurred within a relatively brief average interval of 7.22 ± 1.64 months. These data strongly suggest that repeated hormonal stimulation over short periods can compromise uterine physiology, likely through disruption of luteolysis and sustained progesterone exposure [11,87,88]. Chronic progesterone elevation may reduce uterine contractility and impair the elimination of uterine secretions, resulting in intrauterine fluid accumulation and infertility.

Taken together, our results underscore the need for a critical reassessment of estrus synchronization practices in intensively managed dairy sheep operations. Although such protocols are indispensable for improving reproductive efficiency, there is accumulating evidence that their indiscriminate and repeated use—particularly in vulnerable animal groups—may predispose to significant reproductive pathologies. More conservative approaches, including extended intervals between treatments, reduced frequency of hormonal administration, adjusted dosage of eCG according to the sheep breed, the season of the year which the mating occurs, the animals’ weight, and, as alternative to eCG option, the adoption of eCG-free regimens (e.g., prostaglandin-based synchronization), could mitigate the risk of complications. Tailoring these strategies to account for age, parity, and physiological status—especially in hoggets—would likely improve both animal welfare and farm-level reproductive performance.

For farmers, an individualized approach to estrus synchronization could reduce reproductive disorders like vaginal prolapse and hydrometra, improving flock productivity and welfare. Policymakers should encourage research into alternative reproductive methods, such as non-hormonal synchronization protocols, to support animal welfare and the sustainability of the dairy sheep industry.

This study’s limitations include its focus on farms in Thessaly, Greece, and a specific genotype. Future research should expand to other breeds and regions and examine the long-term effects of hormonal treatments on animal health and reproductive performance. Exploring alternative synchronization methods, genetic factors, and the role of nutrition and environment in reproductive health will be crucial for advancing sustainable reproductive management practices.

## 5. Conclusions

This study highlights reproductive challenges in estrus synchronization protocols for Lacaune-crossbred sheep managed with intensive systems. Hoggets carrying multiple fetuses are at a significantly higher risk of vaginal prolapse. Given that eCG increases the likelihood of multiple ovulations, its routine use in underweight or young animals should be re-evaluated to minimize this risk. Additionally, the increase in hydrometra incidence after repeated synchronization treatments suggests a link to prolonged progesterone exposure and its effects on uterine function.

These findings underscore the need for a tailored approach to estrus synchronization. Limiting hormonal treatment frequency, extending recovery periods, adjusting protocols based on age and physiological status, and using eCG-free regimens in hoggets could enhance reproductive management in dairy sheep populations.

## Figures and Tables

**Table 1:** Summary of study population characteristics.

Parameter	Mean ± SD/Frequency (%)
Adult body weight (kg)	70.0 ± 4.1
Annual milk yield (kg)	420 ± 42
Total reproductive females	97,215
Hoggets	14,494 (14.9%)
Ewes (2nd parity)	30.30%
Ewes (3rd parity)	30.60%
Ewes (4th parity)	24.10%

**Table 2:** Prevalence of vaginal prolapse in hoggets and ewes.

Category	Hoggets (n, %)	Vaginal Prolapse in Hoggets (n, %)	Ewes (n, %)	Vaginal Prolapse in Ewes (n, %)
Total Pregnant Animals	14,494 (100%)	135 (0.93 ‡^a^)	71,445 (100%)	243 (0.34 §^a^)
Singleton Pregnancies	3154 (21.76%)	5 (0.16 ‡^b^)	14,245 (19.94%)	24 (0.17 §^b^)
Twin Pregnancies	6275 (43.29%)	69 (1.10 ‡^c^)	31,740 (44.43%)	105 (0.33 §^a^)
Triplet Pregnancies	5065 (34.95%)	61 (1.20 ‡^c^)	25,460 (35.64%)	114 (0.45 §^c^)

‡, §: Prevalences between hoggets and ewes followed by different symbols are significantly different (Chi-square test, *p* < 0.0001). ^abc^: Within hoggets or ewes, prevalences in the same column followed by different letters differ significantly between categories (ANOVA, *p* < 0.0001).

**Table 3:** Prevalence of hydrometra in ewes.

Category	1st E.S. (n, %)	2nd E.S. (n, %)	3rd E.S. (n, %)	4th E.S. (n, %)
Treated	80,250 (100%)	5542 (100%)	585 (100%)	146 (100%)
Pregnant	74,408 (92.72 ^a^)	4865 (87.78 ^b^)	403 (68.89 ^c^)	62 (42.47 ^d^)
Non-Pregnant †	5842 (7.28 ^a^)	677 (12.22 ^b^)	182 (31.11 ^c^)	84 (57.53 ^d^)
Hydrometra	0 (0%)	0 (0%)	3 (0.51 ^a^)	18 (12.33 ^b^)

†: Includes cases of hydrometra. ^abcd^*:* Prevalences in the same row followed by different letters are significantly different (ANOVA for pregnancy and non-pregnancy; Chi-square test for hydrometra, *p* < 0.0001).

## Data Availability

Data are contained within the article.

## Abbreviations

The following abbreviations are used in this manuscript:

PDO	Protected Designation of Origin
ES	estrus synchronization
eCG	equine chorionic gonadotropin
